# Understanding the Challenges of Intensive Care Staff in Communicating With Patients and Patients’ Families During the COVID-19 Crisis: A Qualitative Exploration

**DOI:** 10.7759/cureus.40961

**Published:** 2023-06-26

**Authors:** Manjulika Vaz, Carol D'Silva, Bhuvana Krishna, Priya Ramachandran, Moses C D’Souza, Lavina Mendonca, Padmalatha Raman

**Affiliations:** 1 Health Humanities, St. John's Research Institute, St. John's Medical College, Bangalore, IND; 2 Critical Care Medicine, St. John's Medical College Hospital, Bangalore, IND; 3 Pulmonary and Critical Care Medicine, St. John's Medical College Hospital, Bangalore, IND; 4 Anesthesiology and Critical Care Medicine, St. John's Medical College Hospital, Bangalore, IND; 5 Nursing, St. John's Medical College Hospital, Bangalore, IND; 6 Anesthesiology and Critical Care Medicine, Prakriya Hospitals, Bangalore, IND

**Keywords:** critical care nurses, qualitative research, humane medical practice, healthcare professionals and icus, pandemic challenges, mobile phones, ppe, communication, icu, covid-19

## Abstract

Background

Coronavirus disease 2019 (COVID-19) isolation protocols in India restricted family members of COVID-19 patients from visiting them in hospitals and in intensive care units, especially during the peak of the pandemic. This along with the elaborate personal protective equipment (PPE) created challenges for intensivists and nurses in COVID ICUs in effectively communicating with patients and patients’ families, especially in shared decision-making processes.

Methods

This article is the outcome of a qualitative study using in-depth one-on-one interviews with 10 intensivists and four intensive care nurses in two teaching hospitals in Bengaluru, South India. Each participant, purposively selected till data saturation was reached, had spent extensive periods of time in a COVID ICU during both COVID-19 waves in 2020 and 2021. A framework of descriptive phenomenology led to the design of the study in which varied experiences and insights of participants were captured using an interview guide to understand their lived reality. The interviews were conducted online or in person between July 2021 and October 2021 and were audio recorded and transcribed verbatim. Coding of transcripts using the NVivo 12 (Burlington, MA: QSR International Pty Ltd) software helped with the thematic analysis. This was guided by interpretive phenomenological methods that derived meaning from participants’ life experiences.

Results

Four themes involving challenges in effective communication in the COVID ICU emerged as follows: physical barriers, emotional and mental stressors, infrastructural challenges, and ethical and moral dilemmas. Sub-themes included personal protective equipment as a barrier, reduced energy levels, and isolation of family from patients under the domain of physical challenges; fears of the unknown, handling death of patients in isolation, and the frustrations of families were challenges under the emotional and mental domain. Infrastructural/systemic challenges included poor connectivity and insufficient mobile phones, and the absence of rules to handle interruptions. Privacy breaches, taking consent over the phone, end-of-life discussions, and medico-legal risks emerged as the subthemes under the domain of ethical and moral challenges. A mobile phone communication policy specifying usage times and operating methods, a mandatory communication and counseling training module for intensivists and intensive care nurses, and a set of protocols for highly restrictive, intensive care units in pandemic situations were recommendations and lessons learned.

Conclusions

The lack of face-to-face interactions was a serious barrier to communication between ICU staff and patients and their caregivers. It had a bearing on trust levels and had emotional and ethical consequences for healthcare teams to handle. Opportunities for self-care, venting of anxiety and distress, and opportunities to celebrate and reward special efforts and cooperation between consultants, residents, nurses, and technicians in stressful environments like a pandemic ICU were important to sustain empathy and keep care and communication humane.

## Introduction

Effective communication with family members of patients in intensive care units (ICUs) is key to effective decision-making, improving trust with ICU physicians, patient satisfaction, psychological well-being, and maintaining a good rapport [1]. Effective communication has, however, been a challenge during the coronavirus disease 2019 (COVID-19) pandemic. Staff were required to wear personal protective equipment (PPE) which apart from making doctors and nurses hard to identify, reduced the ability of those in PPE to hear and to be heard clearly. COVID-19 isolation protocols dissuaded family members from visiting patients in the ICU to prevent the further spread of the infection [2]. Very often family members tested positive for COVID-19 and were either isolated at home or in a hospital ward. For all these reasons, ICU physicians were unable to meet face-to-face with family members. In addition, ICUs were under tremendous pressure; the demand for beds often exceeded the capacity in most countries, including India. As of January 1, 2022, India had reported over 35 million cases of COVID-19 with close to 0.48 million deaths attributed to it. The mortality rates were significantly higher in the second wave of COVID-19 [3].

In pre-pandemic times, there were a fixed number of ICU beds, manned by a team of intensivists and nurses in a 1:1 or 1:2 ratio for each patient along with other allied health specialists. Patients’ caregivers were counseled through face-to-face discussions about the patient’s progress, treatment options, need for medical/surgical procedures, futility of care, etc., usually in a private room or area designated for counseling. At the peak of the pandemic, however, ICU beds had to be rapidly increased to cope with the rapid surge of cases, with the pooling of nurses and doctors across the hospital to care for these patients. ICUs had to contend with limited availability of resources, rapidly changing treatment protocols, the rapid deterioration of patients, and high mortality. These unprecedented circumstances had resulted in extreme distress in ICU healthcare providers and patients and their families [4]. Lack of face-to-face interactions between doctors and families of patients, and between patients and their loved ones appears to have exacerbated this distress [5].

To overcome the limitations in communication with families and between patients and their immediate family members, various innovations, such as online video conferencing and mobile phone conversations, facilitated virtual counseling and virtual visits [5,6].

In India, these challenging circumstances compounded the experiences of healthcare workers (HCW), both doctors and nurses in COVID ICUs, in effectively communicating with patients and their families. This qualitative study was designed to explore this novel phenomenon and the lived experiences of HCWs during the COVID pandemic in relation to patient and patient family communication. Specifically, it aimed to understand the challenges in communication faced by doctors and nurses in designated COVID ICUs including the lessons learned at a personal level and at the level of the ICU in using novel ways of communication, towards future application, training of residents, and policy development. Preliminary findings as an abstract were presented orally at the research day event at the institution of the authors in May 2022.

## Materials and methods

This study followed a qualitative research approach guided by a framework of descriptive phenomenology in which varied experiences and insights of participants were captured to understand their lived reality. The researcher's position was as an outsider journeying with participants to become an insider and used a combined process of participant - researcher-led interpretation of narratives with inductive logic. The choice of this approach was because “qualitative studies could provide important insights regarding the effectiveness and limitations of the different communication strategies” [5]. The non-positivist, interpretivist paradigm of qualitative research has also been found to be of value in the critical care setting of hospitals [7].

The study sites were two tertiary teaching hospitals in Bengaluru, India, chosen based on the interest shown by intensivists in this topic. Both were private, non-funded, tertiary care hospitals and COVID-19 nodal centers for the city of Bengaluru. The study was reviewed and granted ethical approval by the institutional ethics committees (IEC) of both institutions (IEC study ref. no. 159/2021 and IEC/SMH/PS0316/IEC/July 16/2021). Participation was voluntary with individual written consent. No incentives were offered to participate. Sampling was purposive to include intensive care doctors and nurses who had spent at least 10 shifts (each shift of 6 hours=60 hours of duty) in the COVID ICU, across levels of seniority, departments of deputation, and gender. Exclusion criteria included those who were physically unwell and those unwilling to participate.

Recruitment of participants started in July 2021 and ended in October 2021. Following written informed consent, in-depth interviews with individual participants were conducted by a researcher, a social scientist, well versed in qualitative research methods (MV). Due to restrictions in entering COVID ICUs, these interviews were conducted online using the platform Microsoft Teams (Redmond, WA: Microsoft Corporation), following an explanation about the purpose of the study, addressing questions on the study, voluntary nature of participation, negotiating a convenient time, and receiving their written informed consent. An open-ended interview guide that was pilot tested was used and with the permission of the participant, the interview was recorded. Each interview lasted an average of 60 minutes and was conducted in English (the common language of communication). These were all individual interviews, with nine of the 10 interviews with doctors done online and all the interviews with nurses done offline. Data collection continued until data saturation was reached, i.e., no new data emerged. Sample questions are provided in Table 1. A copy of the full interview guide is available with the first author on request.

**Table 1:** Sample of questions from the interview guide. COVID-19: coronavirus disease 2019

Domains	Questions
Work life in COVID ICU	Are there differences between pre-COVID times in the ICU and now? Can you explain what they are?
Communication issues	Are there communication challenges that you faced in the COVID ICU? What are they and could you suggest why they occurred?
Novel methods and your experiences	Were there changes in communication practices during COVID-19? Can you describe the different methods used, how they were used in different circumstances, and your experiences with them?
Suggestions and feedback	Could you share your thoughts on how communication can be improved in these times considering all these challenges?

Audio recordings were transcribed verbatim and manually by an external professional agency, with whom a confidentiality agreement is in place. Interpretative phenomenological analysis (IPA), based on Heideggerian hermeneutics was used to find meaning in people’s experiences [8,9]. The primary analyzer was the first author (MV) who was an outsider to the ICU setting. Using the Husserl method bracketing of former knowledge of ICU care experiences was relatively easy for her with interpretation being led by participant experiences. She carried out inductive coding of the transcripts after multiple rounds of reading and re-listening to the audio recordings. The first author’s embeddedness in the field of bioethics led to the interpretation of moral and ethical encounters in the doctor-patient and patient family exchanges. This fore-conception has influenced the interpretation of data. Coding was aided by the NVivo 12 software (2018) version 12 (Burlington, MA: QSR International Pty Ltd.) (https://www.qsrinternational.com/nvivo-qualitative-data-analysis-software/home). Clustering of codes into categories followed by the arrival of larger themes took place in consultation with the second author (CD), an intensivist with nine years of experience and positivist medical training, to arrive at the first level of thematic analysis. This first-level analysis was presented and discussed with members of the critical care unit, both doctors and nurses, both participants and non-participants of this study. Their feedback was received which helped in further interpretation of themes. Multiple investigators from medical, surgical, and respiratory critical care, along with critical care nursing helped to sharpen these insights, bring in reflexivity and further interpretations. The Consolidated Criteria for Reporting Qualitative Research (COREQ) requirements were followed to ensure the trustworthiness of the data, its interpretation, and reporting [10].

## Results

The participants of the study included 10 doctors and four nurses. The doctors were primarily intensivists who worked either in a medical, surgical, or respiratory ICU. The mean age of the respondents was 30 years. Four of the 10 doctors (40%) were males while all the nurses were female, a prevalent situation in most Indian hospitals. On average, each participant had worked in a COVID ICU exclusively for five weeks in total in the first wave of COVID-19 (March-September 2020) and seven weeks in the second wave of COVID-19 (March-June 2021). Details of the respondents in our sample are mentioned in Table 2.

**Table 2:** Characteristics of the participants. MICU: medical ICU; SICU: surgical ICU; RICU: respiratory ICU

Respondents	Doctors (n=10)	Nurses (n=4)
Male/female	4/6	0/4
Primary department: MICU/SICU/RICU	5/3/2	-
Age range in years: 40+/30-39/20-29	3/7/0	0/0/4
Average duty shifts in weeks (1st wave/2nd wave)	5/7	5/7

**Figure 1:**
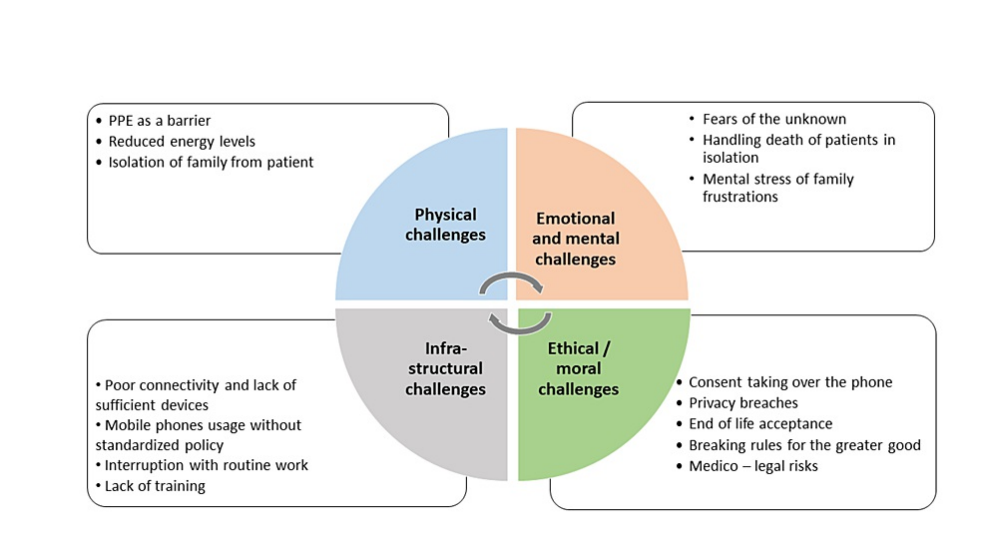
Themes and sub-themes of challenges experienced in communications in COVID ICUs. PPE: personal protective equipment; COVID: coronavirus disease

There are four superordinate themes that emerged from the analysis which are physical challenges, emotional and mental challenges, ethical/moral challenges, and infrastructural challenges. Sub-themes of PPE as a barrier, reduced energy levels, and isolation of family from patients emerged under the domain of physical challenges; fears of the unknown, handling death of patients in isolation, and the frustrations of families were challenges under the emotional and mental domain. Infrastructural challenges included poor connectivity and insufficient mobile phones, and rules to handle the interruptions. Privacy breaches, taking consent over the phone, end-of-life discussions, and medico-legal risks emerged as the subthemes under the domain of ethical and moral challenges (Figure 1). The corresponding codes and data are presented in the form of verbatim quotes. These quotes from respondents have the personal identities masked but a numerical code is provided with, additionally "D" or "N" denoting doctor or nurse, respectively, "M" or "F" their gender, and respiratory/medical/surgical ICU (MICU/SICU/RICU) denoting their primary departments.

Physical challenges

The Barrier of PPE Itself 

PPEs were experienced as a communication barrier as they interfered with hearing, identifying people, and depriving touch and body language.

Hearing Challenges

The PPE’s hood over the head, with tightly strapped goggles/protective eyewear affected the participants’ auditory acuity. Those who used respirators had even more difficulty. HCWs within a COVID ICU were forced to speak louder which added to their physical exhaustion as explained by this doctor, “voices were muffled… people could not hear clearly… we had to really raise our voice” (D-09, M, SICU).

Identifying People

This was another problem caused by the PPE with all HCWs appearing alike to the patient, as well as to each other, resulting in some confusion and mix-up of identities. Patient caregivers also expressed difficulty in identifying the HCW talking to them in a video call and required a formal introduction from the caller. Locating a particular HCW within the ICU and identifying one another from a distance was also a challenge. Writing the person’s name on the PPE and getting attuned to voices helped overcome these difficulties.

Touch and Body Language

Its use in non-verbal communication was deprived to some extent by the PPE. While the use of PPE did not completely forbid touching the patients, the use of the double/triple layer of gloves affected tactile perception. As described by one of the doctors, “you were not able to touch or feel the patient… my examination in terms of touching, feeling took a back seat… this was a major issue I felt” (D-01, F, MICU).

Reduced Energy Levels

Physical exhaustion due to long working hours in COVID ICU with PPE, dehydration, odd hours of shifts, increased number of patients, and the need to speak louder. The participants expressed that they just wished to be done with their shifts due to exhaustion. Communication required extra energy and with high levels of fatigue, it was difficult.

Isolation of Families From Patients

A ban on families entering the ICUs or healthcare facilities was the outcome of strict national and state government regulations on the confinement of primary contacts of patients who had contracted COVID-19. In addition, relatives who had traveled from within or outside the country were also COVID-19 suspects and were quarantined.

The challenge experienced by intensivists in the absence of face-to-face communication was difficulty in convincing family members about the condition of the patient. This was particularly hard in explaining medical terms, responding to visual feedback, and lack of comprehension. Acceptance of the rapid deterioration of the patient’s clinical condition, denial of the patient’s condition or prognosis, understanding of escalating ICU costs, and channeling expressions of grief were added to communication challenges. With the switchover from in-person counseling to counseling over a mobile phone or landline, a trust deficit was created between patients’ families and doctors. They were unable to see the progress of the patient and failed to understand why the patient didn’t respond to appropriate treatment resulting in suspicions, casting of aspersions, and emotional and even physical outbursts as expressed here: “… physical presence, actively seeing the patient and what's happening to them actually matters. To many of them, it is a sort of emotional connection, and when they see… there is a higher degree of trust" (D-07, F, MICU).

Another doctor explained, “the last time the family saw the patient, was when the patient walked in with some minimal oxygen support… (there was) very little time to convey to them that, I needed to intubate, and I need to put them on a ventilator. And we know that outcomes are not great, it's difficult for them to accept it because they can't see exactly what is happening" (D-02, F, MICU).

Mental and emotional pressures

While at the best of times, an ICU is a fast-paced and challenging environment, the pandemic posed greater emotional stress, sometimes irrational emotions and mental dilemmas brought on with the rapid surge of cases. These emotional and mental stressors were bilateral, i.e., affecting the HCW and the patients alike. The following are the sub-themes explaining this theme.

Handling Fears of the Unknown and of Patients

Particularly in the first wave, with minimal knowledge of the natural course of the disease and any proven treatment of benefit, participants were fearful and wished to stay away from closed ICUs to avoid contracting the disease and risk transmitting it to their loved ones at home. Compared to the first wave, the second wave saw younger patients succumb to the disease, which hit the doctors and nurses on duty hard and increased perceptions of fear. In the absence of families inside the COVID ICUs, HCWs were the only human contact with patients at the time. With louder communication between HCWs, the responses to a crashing patient, perceptions of death, and the uncertainty of what to expect caused patients to relay their fear to nurses particularly. As this 23-year-old nurse reasoned, “… because they cannot see relatives, … and they know many people are dying and have thoughts of death, they turn to us…” (N-04, F, MICU). A doctor explained similar distress, “it was very tough for the family because having a sick member of the family in hospital is traumatic… not being able to see them… not being able to meet your doctor, communicate freely, (even more)..." (D-03, M, MICU).

Handling Emotions of Family Members, Especially on the Death of Patients

While it was the intensivist who handled the phone communication on clinical events and procedures to the next of kin, it was the nurses who usually faced the emotional responses from family members with regard to not just the clinical condition of the patient but to the food intake of their near one, their pain or sense of comfort, and also on financial matters. Handling grave news of a patient’s sudden deterioration or death was particularly difficult and took an emotional toll. Post-death protocols made it mandatory for the bodies of patients who died due to COVID-19 to be sealed in a certain way thus denying family members a final goodbye. It was the nurse who was the surrogate family member and made the final connect between the patient and the family. The emotional toll is apparent in these words, “… when a patient is deteriorating, relatives cannot see… cannot know what is happening… most challenging was at the time of death. Packing the body was an important thing. There was too much work… the chain of duties, consoling family also, …. too much stress” (N-03, F, MICU).

In the second wave, multiple members of the patients’ families were admitted to different hospitals. Doctors participating in this study expressed the pressure they felt when they were aware of the demise of a patient’s close family member but had to refrain from revealing it till the patient got better. A female doctor explained, “…also, another challenge we have had (was) that a family member is dead somewhere and we had to hide that from the patient…” (D-01, F, MICU).

Handling Overwhelming Numbers, High Stress, High Demand on Infrastructure, and the Mental Stress of Family Frustrations

Many HCWs contracted COVID-19 themselves and had to remain isolated. The work pressure expressed by participants, especially the nurses who were living away from home, included the inability to take leave, coping with stressful working hours, and the inability to go home to their families. Those intensivists who felt strongly about the part played by communication in the effectiveness of their role, had this to say, “the bulk of our stress comes from managing communications… 25% is clinical work and 75% counseling work… we even got used to the PPE. But we could never elevate the complete communication with the family” (D-07, F, MICU).

Infrastructural challenges of mobile phones and video devices in filling in the communication gap

Mobile phones are not part of the usual communications in day-to-day counseling of ICU patients. During the pandemic, however, its benefits were experienced in multiple ways. They served as a good alternative to maintain contact between patient and family, as well as the doctor and patient’s family. The use of video calls especially helped to reaffirm the patient’s condition as explained by one of the doctors, “it helped in particular to explain procedures such a proning, a life-saving technique in hypoxic patients, which would be difficult for the lay public to comprehend with just words” (D-03, M, MICU).

Online messaging platforms such as WhatsApp served an important purpose including requesting necessary case records as well as official essential documents. It also helped the ICU send out periodic case summaries. The nursing staff too found it convenient to convey and receive information and essential documents pertaining to treatment and financial subsidies. Audio calling was done for initial information and video calling for explaining procedures, showing patient monitors, or in situations when patients were too sick to speak. While these communication devices had several advantages there were challenges too. The challenges included the following problems.

Poor Connectivity and Lack of Devices

Connectivity problems, weak internet signal, unavailability of phones or smartphones that support video calls, call drops or poor voice quality over the call, and families not being available to pick up calls when the doctor had time allotted for counseling were constant niggling problems.

Interruption With Routine Work

The phone would ring all the time, interfering with the HCWs’ routine, resulting in frustration as expressed here, “attenders, they want to know the condition every hour, every half an hour...” (D-05, F, RICU); “… because there are 30 patients in your ICU and everyone is constantly calling for everyone, you start to ignore the phone after one point of time, or just cut the calls” (D-03, M, SICU).

Mobile Phones Usage Without Standardized Policy and Specific Training

It became a transactional, insensitive mode of communication, sometimes bordering on a lack of propriety when the same device was used by someone to convey a matter related to an outstanding bill payment followed immediately by a call on a delicate matter, such as the intubation or deterioration of the patient, as explained here, “another issue we faced is that the same phone was used to communicate about the bills… someone would call and say the bill has been sent to you, please see the bill and then the doctor immediately after that calls and says, your patient is dying...” (D-06, F, MICU).

There were instances of misinterpretation of information due to the minimal time available to use these devices, and absence of body language messaging leading to information gaps, doubts, and mistrust. Audibility was at times the reason because the physician being masked and at times it was the inability to handle all questions and anxieties over the electronic device. Discussing one’s relatives’ health over the phone with a “masked” stranger, without being formally introduced and without following communication niceties, was seen to be a starting point of mistrust. Language barriers would come in the way when the HCW and the patient’s family spoke different languages and using language translators or social workers was not possible over the phone, as there were limited staff permitted into a COVID ICU. Group or conference video or audio calls that these devices supported meant that the HCW was often talking to strangers which included distant relatives, doctors, or intensivists from other hospitals and so on. This was considered inappropriate and sometimes even deceptive.

Ethical and moral challenges

An underlying challenge resulting from the lack of face-to-face communication with the next of kin and the use of electronic devices for communication was the ethical and legal issue of informed consent and privacy. Handling end-of-life acceptance, joint decision-making, private interest versus public good and medico-legal risks were additional moral burdens faced by HCWs in these contexts.

Consent Taking

For every discussion and decision made by the next of kin or legal guardian of the patient related to intubation or other procedures, verbal consent was taken. However, this was noted in the case sheet without a signature and without an audio recording of the oral phone conversation. This created a fear of medico-legal complications of not having written informed consent for the many procedures done on the patients. Explaining “central venous access” or “arterial line,” its risks, and complications were far more difficult on the phone. It was also said that one really did not know who was giving consent on the other end of the phone. As these respondents said, “consenting became a major issue… they can say - I never gave consent if it’s not recorded... legally (they’re right), if they want to make a problem later” (D-06, F, MICU). “We take this (consent) telephonically and we document, but definitely the quality of the consent will not be the same. …it’s harder to ensure or to confirm whether they’ve understood everything or not” (D-02, F, MICU).

The other challenge posed by the electronic communication device was that the communication between HCW and the family could be recorded by the patient’s family and possibly used against the doctors, “many attendants recorded all that we have spoken to them and things like that. Whereas, in the non-COVID ICU, we wouldn’t obviously allow that… we’d be far stricter” (D-06, F, MICU).

Privacy

Violation of personal privacy was an unintentional fallout because of the need to talk loudly while counseling due to wearing of PPEs. This resulted in other patients overhearing the details of the patient’s condition. Similarly, despite all precautions, such as drawing curtains, patients were possibly in compromising positions in video calls. An invasion of personal space and time was felt at moments that were intimate and private for the patient and their families, such as the following sharing by an intensivist and a nurse respondent, “before we intubate someone, I call the family and say, you can spend a couple of minutes with the patient, and we walk away and give them that privacy so they can talk. But in the COVID situation, we became privy to a lot of these (conversations)… probably the last interaction with the family, and you become part of that interaction… ” (D-07, M, MICU).

End-of-Life Acceptance

Initiating end-of-life care discussions for terminally ill patients are always a challenge and involve many meetings with close family members, taking time to help them reach a decision. This became far more tedious over a phone call and with the pressures of time, especially in the second wave where mortality was high.

Handling Dilemmas Following Rules Versus Doing What Is Possibly in the Best Interest of the Patient

The challenge between adhering to the rule made in the interest of the public versus breaking the rule in the interest of an individual distressed or a dying patient was also an ethical conundrum. A conflict between the existing rule prohibiting families to visit patients and what was considered in the best interest of the patient to allow them in was a recurring dilemma. Family members would express at least once during counseling, for permission to visit their patients in the COVID ICU. As this intensivist expressed frustration, particularly in the case of elderly or dying patients, “ …it bugged me at one point of time… should I allow them in, should I not… I sort of in my head made these ground rules that if this patient is about to die, if this patient is really anxious and I know that no amount of nursing care or drugs were going to calm them down… (then) I should allow them in… ” (D-06, F, MICU).

Medico-Legal Risks

Each of the above ethical challenges had medico-legal implications which could be used against the HCWs in the future, thus making them vulnerable. Some nurses and doctors shared instances of threats faced by them. As one doctor quoted "Families found it a huge problem to accept a patient's rapid deterioration and this resulted in outlandish accusations... " (D-01, F, MICU).

Future directions

The communication difficulties encountered in the COVID ICU led to both personal and professional development lessons at the level of an ICU, especially in readiness for a future exigency. Table 3 provides the derived lessons and recommendations for the future. They cover policies, training protocols, and hospital management decisions. These lessons, derived from the lived experiences of the study participants, have universal applicability and benefit. Having clear protocols to streamline timings of calls, save a patient’s next of kin’s phone number on a device at admission and ensure its deletion post-death/discharge, decide on the suitability of voice calls over video calls, allow for group calls with other specialists, such as social workers, translators, mandatary processes and training for consent taking over phones and awareness of privacy laws, counseling over a phone in emergency/pandemic situations, breaking bad news, keeping the communication simple, and policies for visitation in pandemics, not only would enhance efficiency and avoid legal repercussions but would make the ICUs functioning more humane and reduce certain levels of moral distress.

**Table 3:** Lessons towards strengthening communication systems during pandemics.

Themes	Lessons learned/Issues identified by HCWs
Mobile phone communication policy	Clear process and responsibility of saving next of kin number. Fixed time for attending calls, sequence of calls to family to be fixed. For example, start with doctor’s counseling, followed by billing and finance related. Need to ask permission before disclosing patient information to unsaved numbers. Use audio calls for routine information, and restrict video calls for counseling. Clear protocol for bringing in translators, and social workers on counseling calls. Clear protocol on recording consents on phone and saving procedure. Clear protocol for ensuring the privacy of patient and patient information. Delete phone numbers on death or discharge.
Communication and counseling training for intensivists and ICU nurses	Communication with patience giving details of uncertainty and rapid deterioration possibilities towards building trust. Truth-telling with balance of reassurance. Learn how to address “silly" and waste of time questions". Being courteous even when stressed and handling emotional stress of death. Importance of clear, simple, empathic language. Handling transference and countertransference. Use ongoing situations, such as global news to help convey patient's condition. Anticipate requirements of consent and prepare the family. Shadow seniors during counseling. Seek feedback and guidance.
Protocols for high restriction, intensive care units in pandemic situations	Pre-ICU admission communication process needs to be rigorous. Detailed handover communication processes at shift change, especially because of leave periods. Intra-team communication processes to avoid mix-ups. Visitation protocols for family members, support groups, and religious persons with testing, fitness, and PPE compliance. Stick to only one to two close relatives for counseling. Repeated counseling in case of deterioration in patient condition. Increased number of personnel, specifically to handle phones, financial matters, counseling support, government-police liaison. Opportunities for inter-team anxiety management, such as bonding/destressing activities. Celebrate and reward-special efforts and immense cooperation between consultants, residents, nurses, and technicians.

A crisis like the pandemic with unprecedented distress in the lives of HCWs and the public alike brought out the best in people. Amid adversity, there were lessons in empathy and opportunities where ethics and medical humanities could be caught and not taught.

In the words of a deeply moved HCW, “If my father was there (in COVID ICU) I would also keep calling… I would feel like that... I’m happy I could spend more time speaking to patients… on behalf of… in place of their families. “… I became close to patient relatives on group video calls… they asked for me to please feed their father… they thank so much” (N-04, F, MICU).

## Discussion

Our study sought to explore the challenges in communication during the COVID-19 pandemic from the HCW perspective. An ICU admission is distressing for patients and their loved ones, however, during the pandemic, the distress was amplified and experienced by both patients and the HCWs. All of them experienced difficulties in coping during this time and communication was an important challenge.

The first theme we identified was the physical barrier due to the PPE. Fatigue and mental exhaustion were repeatedly expressed in our study and have been reiterated in other studies globally and in the Indian context where Agarwal et al. showed that 100% of the participants experienced excessive sweating and 75% of them had fatigue and headaches due to PPE use [11]. The use of PPE, especially with the N95 and elastomeric respirators, affected auditory acuity causing HCWs to raise their voices to communicate. This has been experimentally proven in simulated settings [12]. Identifying people with PPE was also a difficulty. This lack of identity of one’s treating doctor or nurse may have increased fear and anxiety among patients in an already stressful environment. The use of name tags/designation tags has been described in several hospitals and our participants also used this method, particularly in the second wave [13]. The use of hand signals and gestures has been extensively tried to simplify communication with PPE [14]. This technique was, however, not stated by our study participants.

PPE also affected core communication skills as it eliminates the use of body language and non-verbal cues [12]. There is no denying that "touch" which is such an important component of care and healing, and a social bond of sympathy and compassion between two strangers was a challenge in COVID ICUs, primarily due to PPEs. The importance of touch has been emphasized by Dr. Horton who explains how “touch can even convey the idea of survival” [15]. Use of PPE has been seen as dehumanizing due to the loss of the human touch and connection with patients. Patients have experienced a sense of disconnection by not being able to see or hear their doctors or bedside nurse clearly. Touch, in clinical medicine, does not just help in coming to a diagnosis but is a mode of communication, soothing and healing the patient all at once [16]. Appreciating the patient’s pulse, feeling the warmth of the patient’s peripheries to assess their perfusion, or just a warm handshake as a reassuring sign to the patient was hard to do with gloved hands. The participants in the study felt deprived of this key yet taken-for-granted communication tool. The frustration and the impracticality of the PPEs in carrying out ideal communication with patients in ICUs and their families were emphasized in a recent reflection by Venkateswaran et al. from India [17].

The pandemic wrecked healthcare systems across the world by overburdening existing health infrastructure in terms of bed capacity or trained HCWs. HCWs were able to cope only to a certain extent as they experienced fear, anxiety, and turmoil. Their communicative abilities were affected as they were pushed to the limits of their physical and emotional capabilities. Qualitative studies done exclusively among nurses were able to pick this up and describe its impact [18,19]. Burnout of HCWs due to sheer physical and emotional exhaustion was indirectly expressed by our respondents and reflected among a large number of HCWs surveyed during the pandemic using the Copenhagen Burnout Inventory [20].

The fear and vulnerability of patients were heightened due to the absence of their loved ones. The imposing of lockdowns as a universal mandate made it difficult for the entry of family members into ICUs while the patient suffered alone. The mode of communication with families went virtual universally, with the help of phones, smartphones, tablets, etc. While the use of smartphones was a boon, they have been looked upon as “imperfect solutions” [5]. In spite of infrastructural challenges, such as limited mobile phones and internet connectivity issues, they were extremely useful in conveying information, giving a glimpse of the patient to families, and explaining prognosis of patients. However, other qualitative research studies revealed physicians' discomfort with this mode of communication in conveying empathy and building trust, points expressed by our participants too. Similar to the findings of Kennedy et al., in our study phone calls were used to briefly convey the patient’s condition while video calls were used to corroborate clinical perspectives between physicians and families [21]. Lack of trust stemmed from not personally knowing or meeting the family in person. A lack of privacy was an ethical problem as discussions between patients and their family members would be done in the presence of the HCW holding up the phone.

Not allowing relatives into ICUs increased the communication gaps and mistrust between families and HCWs. The process of visiting their loved ones daily, being updated about their condition, and watching their ups and downs in the ICU is an important consideration of openness and trust. It has been found that inadequate time spent counseling family members or inconsistency in information shared has been linked to anxiety and depression in patients’ caregivers [22]. Communication barriers and negative media coverage of COVID-19 only added to the "nocebo effect," worsening trust issues and emotional distress among families and HCWs [23]. "Nocebo effect" is a negative outcome that occurs due to a belief of harm. It is the opposite of the "placebo effect." A cross-sectional survey done in South Asia and the Middle East on visiting and communication policies showed that 35.4% of hospitals allowed family visits of terminally ill patients as part of reaching end-of-life care (EOL) decisions [24]. In our study, a much smaller proportion of family visits was allowed for terminally ill patients. This dilemma between a critically ill patient’s best interest versus public health interest in infection control was troublesome for HCWs.

To address the frustrations and stresses of communication in ICUs, constituting ICU family liaison teams to communicate the daily condition of patients has been suggested as a strategy for better communication [25]. Lessons derived from our study suggest that this requires a fixed time and preferably a single family member to communicate daily. Prior to the pandemic, written informed consent for ICU admission and any invasive procedures were mandated for legal and ethical purposes. The use of audio or audio-visual consent was also practiced in the participating hospitals prior to the COVID-19 pandemic. However, recording audio or video consent using smartphones along with documenting it in the written mode in the case file has been described particularly for biomedical research done during COVID-19 [26]. Maintaining confidentiality of personal and clinical information on these platforms as well as uniformity in drafting electronic consents are important to consider.

A mobile phone communication policy specifying usage times and operating methods, a mandatory communication and counseling training module for intensivists and intensive care nurses, and a set of protocols for highly restrictive, intensive care units in pandemic situations were recommendations and lessons learned. Learning about breaking bad news, staying empathetic even with anxious/frustrated family members, and improving communication skills can help in reducing burnout in ICU physicians [27]. Training on how to speak in a clear and simple tone, maintaining confidentiality, the art of truth-telling, with subtlety, how to address frivolous questions, and giving family time for closure after breaking bad news were also important. Good training in communication has also been seen to address burnout [27]. Opportunities for verbal ventilation and relaxation are also critical for those in critical care, especially in distressing times like a pandemic. It is important to institutionalize self-care of intensivists and all HCWs to ensure their well-being, their retention in these settings, and optimum patient care [28].

Strengths and limitations of the study

The foremost strength of our study was that it gave intensive care doctors and nurses an avenue to talk about experiences that they had not discussed before and had kept suppressed. The non-directive mode of interviews and narrative building was hence a reflective, non-judgmental, and supportive process. They also had the opportunity to use negative experiences and challenges towards formulating lessons for a future pandemic. This was, therefore, in a way, an empowering and healing process. This study has reinforced Luhmann’s social systems theory where two distinct systems, the medical and the family, are structurally coupled and enabled only through reflexive communication [29].

The limitations of the study include that we only sought the healthcare workers' perspectives on challenges in communication without including the family/next of kin's response or their perspectives. And among healthcare workers, we chose only doctors and nurses, with other allied or support staff left out. These are areas of future investigation, especially in fine-tuning appropriate policies before another pandemic outbreak. Being a limited center study in one region of the country, questions on the external validity of the results may be raised but the findings resonate with other critical care and emergency care studies and hence can be extrapolated to other ICU settings in our country.

## Conclusions

For the future, as part of pandemic preparedness, to handle the emotional and ethical robustness of communication systems between HCWs in ICUs and caregivers, there is a need for a set of guidelines and policies. This would include a mobile phone usage policy that ensures systematic entering and saving contact numbers, protocols to record, store and retrieve vital communication, maintain confidentiality and privacy to ensure legal and ethical compliance, and effective care management. An increased number of personnel - specifically to handle phones, financial matters, counseling support, and government-police liaison are required. Visitation protocols and communication training of intensivists will support and streamline communication.

It is important to note that focus on communication practices is not just for nurses as reflected in literature but for all intensivists. Opportunities should be provided for self-care of ICU HCWs, venting of personal and inter-team anxiety and distress, and opportunities to celebrate and reward special efforts and cooperation between consultants, residents, nurses, and technicians. All of this is important to sustain empathy and keep care and the ICU environment humane.
